# Prevalence of multimorbidity in Germany: impact of age and educational level in a cross-sectional study on 19,294 adults

**DOI:** 10.1186/s12889-017-4833-3

**Published:** 2017-10-18

**Authors:** Marie-Therese Puth, Klaus Weckbecker, Matthias Schmid, Eva Münster

**Affiliations:** 1 Institute of General Practice and Family Medicine, University of Bonn, Sigmund-Freud-Straße 25, 53127 Bonn, Germany; 2 Department of Medical Biometry, Informatics and Epidemiology, University Hospital Bonn, Sigmund-Freud-Straße 25, 53127 Bonn, Germany

**Keywords:** Multimorbidity, Socioeconomic status, Age, Chronic conditions, German health update (GEDA) 2012

## Abstract

**Background:**

Multimorbidity is one of the most important and challenging aspects in public health. Multimorbid people are associated with more hospital admissions, a large number of drug prescriptions and higher risks of mortality. As there is evidence that multimorbidity varies with age and socioeconomic disparity, the main objective aimed at determining age-specific prevalence rates as well as exploring educational differences relating to multimorbidity in Germany.

**Methods:**

This cross-sectional analysis is based on the national telephone health interview survey “German Health Update” (GEDA2012) conducted between March 2012 and March 2013 with nearly 20,000 adults. GEDA2012 provides information on 17 self-reported health conditions along with sociodemographic characteristics. Multimorbidity was defined as the occurrence of two or more chronic conditions in one individual at the same time. Descriptive statistical analysis was used to examine multimorbidity according to age and education, which was defined by the International Standard Classification of Education (ISCED 1997).

**Results:**

Overall, 39.6% (95% confidence interval (CI) 38.7%–40.6%) of the 19,294 participants were multimorbid and the proportion of adults with multimorbidity increased substantially with age: nearly half (49.2%, 95% CI 46.9%–51.5%) of the adults aged 50–59 years had already two or more chronic health conditions. Prevalence rates of multimorbidity differed considerably between the levels of education. Low-level educated adults aged 40–49 years were more likely to be multimorbid with a prevalence rate of 47.4% (95% CI 44.2%–50.5%) matching those of highly educated men and women aged about ten years older.

**Conclusions:**

Our findings demonstrate that both, age and education are associated with a higher risk of being multimorbid in Germany. Hence, special emphasis in the development of new approaches in national public health and prevention programs on multimorbidity should be given to low-level educated people aged <65 years.

**Electronic supplementary material:**

The online version of this article (10.1186/s12889-017-4833-3) contains supplementary material, which is available to authorized users.

## Background

Multimorbidity - typically defined as the presence of more than one chronic condition at the same time in one individual - represents a major challenge for health care systems [1]. Compared to people with no or only a single chronic disease, multimorbid people are more likely to need costly long-term medical care with more than twice as many contacts with physicians in the ambulatory care sector per year [2–4]. Multimorbidity is also connected to a large number of drug prescriptions (polypharmacy) [4–6] and more hospital admissions: a recent study in Canada for example showed that 26.9% of people with 5 or more conditions of their study population experienced at least one hospitalization compared to 4.6% of people with only one condition [7]. Moreover, multimorbidity negatively influences functional and cognitive abilities [5, 8, 9], reduces quality of life [5, 10] and is associated with a higher risk of mortality: in a recent review and meta-analysis, the risk of death for people with at least 2 morbidities was found to be 1.73 times higher compared to people without multimorbidity [11].

There is no gold standard for the definition of multimorbidity [12, 13], so prevalence rates vary from 12.9% to 95.1% depending on the number of chronic conditions examined or the population under study [14]. As multimorbidity becomes more frequent with age, the majority of studies examining patterns of multimorbidity in Germany focused on the elderly [15–17]. Less emphasis has been given to young or middle-aged people. In addition to the strong association with age, there is some evidence that prevalence rates also depend on socioeconomic characteristics [14, 17–19]. In a recent study in Yorkshire in England for example, prevalence of multimorbidity by age was strongly associated with deprivation. Li et al. found differences between people living in the least deprived area and people living in the most deprived area of nearly 20% [19], while in Germany only little knowledge on these issues is available [17, 20]. However, specific knowledge on national patterns and effects of multimorbidity is required in order to be able to develop effective prevention measures. Differences in health care and educational systems as well as people’s mentality make it difficult to transfer international intervention and prevention programs to public health measures in Germany.

Using data of the national telephone health interview survey “German Health Update 2012”, the present study is the first study that aimed at determining age-specific prevalence rates of multimorbidity stratified by educational level in German adults.

## Methods

Our secondary data analysis is based on the Public Use File (PUF) of the national telephone health interview survey “German Health Update” (“Gesundheit in Deutschland aktuell”, GEDA 2012) conducted by the Robert Koch Institute [21]. The Robert Koch Institute is a federal institution financed by the German Federal Ministry of Health and is responsible for the research of infectious diseases as well as for analyzing national long-term public health trends [22]. As part of the health monitoring, the cross-sectional survey GEDA 2012 was carried out between March 2012 and March 2013 gathering information about a range of health related topics involving current health conditions and medical history along with sociodemographic characteristics [23]. The target population included nearly 20,000 fluently German-speaking adults who were at least 18 years old and were living in private households with landline telephone. Using a two-stage sampling procedure, the ADM-Sampling-System (ADM = Arbeitskreis Deutscher Markt- und Sozialforschungsinstitute e. V.) based on the Gabler-Häder method [24, 25] was used for the selection at the household level whereas random sampling at the individual level was performed by the Kish selection grid method [26, 27]. In total, 19,294 participants completed the computer assisted telephone interviews (CATI) which corresponds to a cooperation rate at respondent level of 76.7% and a response rate 3 of 22.1% (based on standards of the American Association for Public Opinion Research) [23, 27, 28]. More details on the methodological procedures are presented in the Additional file 1.

The PUF analysed here includes information on survey participants in an anonymous form. Specifically, it provides data on 17 self-reported health conditions including 15 diseases, namely hypertension, coronary heart disease, myocardial infarction, chronic heart failure, stroke, diabetes mellitus, bronchial asthma, any type of cancer, hypercholesterolemia, chronic bronchitis, chronic liver disease, arthrosis, osteoporosis (limited to participants aged ≥50 years), arthritis and depression [27]. Within the survey, participants were asked, for example, “Have you ever been diagnosed with hypertension, also referred to as high blood pressure, by a physician?” and if responding positively, they were asked “Have you been diagnosed with hypertension in the last 12 months?” By responding positively to the second question as well, it was assumed that a participant is currently suffering from hypertension. The same methodology was also used for other health conditions. In addition, data on self-reported chronic low back pain for at least 3 months and an evaluation of obesity based on WHO’s criteria (BMI ≥ 30 kg/m^2^) [29] using BMI values estimated by self-reported body height and weight for each participant are available. To assess current health conditions, prevalence estimates were determined by variables representing 12-month prevalence when provided. Estimates of four diagnoses associated with long-term damages (coronary heart disease, myocardial infarction, cancer and stroke) were based on lifetime prevalence.

Although various definitions of multimorbidity have been employed in the literature, the core of considered morbidities is similar in most studies and the majority is also available within the PUF [13, 30]. We defined multimorbidity by the presence of at least two (≥ 2) of the 17 health conditions in one person at the same time. The PUF contains information on the educational qualification according to the International Standard Classification of Education (ISCED 1997) that has been summarized into low education (level 1, 2), medium education (level 3A, 3B, 4A) and high education (level 5A, 5B, 6). For age-specific analyses, 10-year age groups were used that are given by 18–29 years, 30–39 years, 40–49 years, 50–59 years, 60–69 years, 70–79 years and 80 years or older.

Prevalence rates along with 95% confidence intervals were computed for both the total cohort as well as for subgroups defined by age, sex and level of education. All prevalence rates were weighted according to the standardized weighting factor based on age, sex, level of education and residential region provided by the Robert-Koch Institute in order to correct for any deviations from the German population structure [23]. Additional file 1 represents this in more detail. Additionally, the unweighted overall number of participants in each subgroup (defined by sex, age or education) is presented. Based on logistic regression, adjusted odds ratios (OR) and 95% confidence intervals were computed to further examine associations between multimorbidity and age, sex or level of education. All analyses were performed using IBM SPSS Statistics (version 22) [31] with the complex sample module and R (version 3.1.0) [32].

## Results

The analyses included data of 19,294 respondents with roughly the same proportions of men and women (48.3% men and 51.7% women). Sociodemographic characteristics of the study population are summarized in Table 1. Almost all age groups were equally represented; only the proportion of adults aged 80 years and older was lower. More than half of the participants had an educational qualification within the medium ISCED category while fewer participants had a qualification within the lowest or the highest category.

**Table 1:** Sociodemographic characteristics of the study population (GEDA 2012)

	n (%^a^)	Percentage with Multimorbidity (95% CI)	Mean number of diagnoses (95% CI)	Median number of diagnoses
All participants	19,294 (100)	39.6 (38.7–40.6)	1.6 (1.6–1.7)	1
Sex				
Male	9318 (48.3)	37.3 (36.0–38.7)	1.5 (1.5–1.6)	1
Female	9976 (51.7)	41.8 (40.4–43.1)	1.8 (1.7–1.8)	1
Age groups (years)				
18–29	2643 (16.2)	7.0 (5.9–8.3)	0.4 (0.3–0.4)	0
30–39	2242 (15.0)	17.2 (15.1–19.5)	0.7 (0.6–0.8)	0
40–49	3665 (19.7)	27.7 (25.7–29.7)	1.1 (1.0–1.2)	1
50–59	3592 (17.4)	49.2 (46.9–51.5)	1.9 (1.8–2.0)	1
60–69	3325 (13.0)	61.7 (59.3–64.1)	2.5 (2.4–2.6)	2
70–79	2936 (14.1)	72.9 (70.4–75.2)	3.1 (3.0–3.2)	3
80+	891 (4.7)	77.5 (73.2–81.3)	3.5 (3.2–3.7)	3
Level of education				
High	8098 (24.1)	31.9 (30.7–33.1)	1.3 (1.2–1.3)	1
Medium	9812 (55.4)	40.1 (39.0–41.3)	1.6 (1.6–1.7)	1
Low	1358 (20.6)	47.4 (44.2–50.5)	2.1 (1.9–2.2)	1
Number of self-reported diagnoses				
0	7043 (37.9)			
1	4349 (22.5)			
2	2899 (14.3)			
3	1929 (9.6)			
4	1254 (6.2)			
5	795 (4.0)			
6	474 (2.5)			
7	270 (1.4)			
8+	281 (1.6)			

^a^Weighted results to represent the adult population in Germany; Level of education: Missing data for 26 participants

The number of self-reported morbidities in one person at the same time varied from 0 to 13. In total, 62.1% (95% CI 61.2%–63.0%) of men and women had at least one of the 17 chronic health conditions and 39.6% (95% CI 38.7%–40.6%) of the adult population were multimorbid with only small differences between men (37.3%, 95% CI 36.0%–38.7%) and women (41.8%, 95% CI 40.4%–43.1%).

**Fig. 1:**
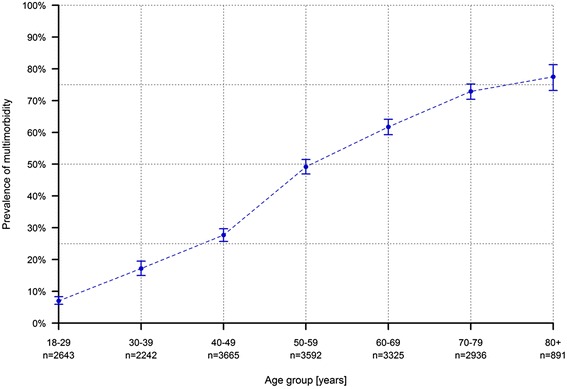
Age-specific prevalence of multimorbidity with 95% confidence intervals

The proportion of multimorbid adults increased considerably with age resulting in an S-shaped curve (Fig. 1). The prevalence of multimorbidity was still lower than 10% among young people (18–29 years old) whereas already more than a quarter (27.7%, 95% CI 25.7%–29.7%) of the people between 40 and 49 years of age were multimorbid. Nearly half (49.2%, 95% CI 46.9%–51.5%) of the adults aged 50–59 years had two or more chronic health conditions and by the age of 80 years, the prevalence rate had grown up to 77.5% (95% CI 73.2%–81.3%).

**Fig. 2:**
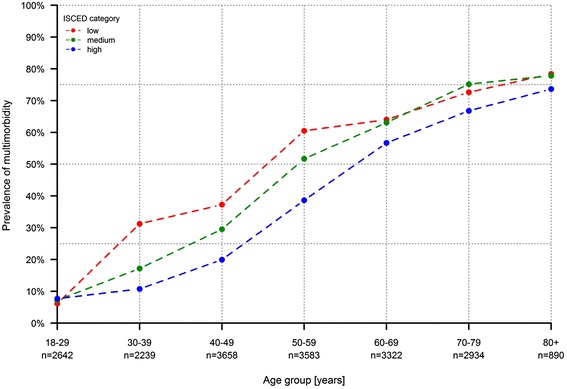
Age-specific prevalence of multimorbidity by ISCED category

Regarding the level of education, people with a lower educational level showed higher rates of multimorbidity compared to those with a higher educational level. Specifically, 31.9% (95% CI 30.7%–33.1%) of people with an educational level of the highest category had two or more chronic conditions whereas nearly half (47.4%, 95% CI 44.2%–50.5%) of the low-level educated people were multimorbid. The association between age-specific prevalence rates of multimorbidity and the level of education is illustrated in Fig. 2. As demonstrated there, the S-shaped curves for prevalence by age varied with education: while prevalence rates for young people (18–29 years old) and elderly people (≥ 60 years old) were similar, there were substantial differences between the three educational levels among middle-aged men and women (30–59 years old). Of note, the curve of the lowest educational level had a steeper slope leading to a considerable shift to the left. As a result, adults aged 40–49 years with a low educational qualification showed prevalence rates equivalent to highly educated adults at least ten years older. Furthermore, for people aged 60–69 years with a high educational qualification, the prevalence of multimorbidity was still lower than for low-level educated people about 10 years younger (50–59 years old).

Age and level of education showed a significant association with the odds of being multimorbid (Table 2). In particular adults with a low or medium level of education had higher odds of being multimorbid than highly educated adults (Adjusted OR (low vs. high) 1.9, 95% CI 1.5–2.2; Adjusted OR (medium vs. high), 1.5, 95% CI 1.4–1.7). Using 18–29 year old adults as reference, the odds of being multimorbid increased with each additional age group, too (Table 2).

**Table 2:** Odds ratios (OR) estimated from logisitc regression for multimorbidity by sex, age and level of education

	Unadjusted OR	95% CI	Adjusted OR	95% CI
Sex				
Male (ref.)	1.0		1.0	
Female	1.2	1.1–1.3	1.0	0.9–1.1
Age groups (years)				
18–29 (ref.)	1.0		1.0	
30–39	2.8	2.2–3.5	3.1	2.4–3.9
40–49	5.1	4.1–6.3	5.6	4.5–7.0
50–59	12.9	10.5–15.9	14.3	11.6–17.7
60–69	21.5	17.4–26.5	23.3	18.8–29.0
70–79	35.9	28.8–44.7	37.2	29.7–46.6
80+	46.0	34.2–61.9	45.2	33.5–60.9
Level of education				
High (ref.)	1.0		1.0	
Medium	1.4	1.3–1.5	1.5	1.4–1.7
Low	1.9	1.7–2.2	1.9	1.5–2.2

## Discussion

The underlying study examined prevalence rates of multimorbidity with regard to age and level of education based on data of the adult residential population in Germany. Multimorbidity is a common issue within Germany that is not limited to the elderly (aged 65 years and older) and already shows prevalence rates >50% in younger age groups, especially in low-level educated adults. In addition to the expected association with age, prevalence rates of multimorbidity differ considerably between the three levels of education. Low-level educated middle-aged adults are more likely to be multimorbid with prevalence rates matching those of high-educated men and women aged at least ten years older.

In general, the lack of a standard definition of multimorbidity limits the comparison of different studies on multimorbidity. Results are usually strongly dependent on the definition of the population under study (e.g. statutory health insurance data or focus only on elderly people), on the number and selection of medical diagnoses and on the choice of a “threshold” describing the number of morbidities that have to be present in one person in order to be considered as multimorbid [13, 30]. Nevertheless, our results agree well with those of other studies on multimorbidity. For example, in a previous GEDA study of 2009, the prevalence rates of multimorbidity defined as two or more conditions in one person at the same time were 43.9% (women) and 36.3% (men), respectively, compared to 41.8% (women) and 37.3% (men) in the present study. Although GEDA 2009 assessed information on 22 health conditions across five age groups only, the prevalence rates for men and women increased with age comparably to the rates of GEDA 2012 [20]. Specifically, the prevalence of multimorbidity rose up to 74.2% for men and 81.7% for women aged 75+ years [20]. In another German cross-sectional study based on claims data, patterns of multimorbidity were evaluated among policy holders aged 65 years and older [15]. The analyses included a list with 46 morbidities comprising all frequent somatic and psychic disorders. Defining multimorbidity as the presence of at least two morbidities, the prevalence rate for adults aged 65+ years was estimated to 73% [15] in comparison to 71.2% in the current study. Patterns in prevalence relating to socioeconomic characteristics are also in line with findings from two cross-sectional analyses in England and Scotland [18, 19]. Barnett et al. examined age-specific prevalence of multimorbidity in Scotland by including 40 different morbidities and evaluating socioeconomic differences by the deprivation of the area in which a patient lived. While only 23.2% in total of the Scottish patients under study had two or more concurrent morbidities (compared to 39.6% in the current study), age-specific patterns with regard to socioeconomic deprivation were similar to those obtained in the present study supporting the description of S-shaped curves as illustrated by Fortin et al. [33]. Specifically, middle-aged people living in the most deprived areas are more likely to be multimorbid with prevalence rates matching those of people living in the most affluent areas aged 10–15 years older. This matches our findings of differences between low-level and high-level educated middle-aged adults causing a shift of the corresponding s-shaped curves. Results of the recent Yorkshire Health Study survey showed that 37.2% [19] of all participants were multimorbid in accordance with 39.6% in the present study. Nearly half (45.7%) of the participants from the most deprived areas had at least two or more of the included 13 health conditions [19], that is comparable to our result of 47.2% for adults with a low educational qualification.

There is a chance that prevalence rates of multimorbidity are under- or overestimated for several reasons, although we cannot determine the direction and quality of it. As the analyses were based on secondary data, only a limited selection of medical diagnoses was available. In particular, prevalence estimates may be downward-biased by not including other relevant chronic conditions such as chronic gastrointestinal diseases. All the details on the different diagnoses are based on self-reported health conditions. Although all participants were asked whether medical diagnoses were made by a physician, information on health conditions were not clinically verified and may be biased as a consequence of misclassification (recall bias/reporting bias) [23]. Only people living in private households were interviewed, people living in nursing homes, for example, could not be contacted. The survey was also limited to people with landline telephone, hence results may be biased by not including households with mobile phones only [23]. As the interviews were carried out in German, people had to speak and understand German [23], so marginalized groups such as migrants could not be regarded [27]. Moreover, people with a low educational qualification agreed less often to participate in the telephone interview than medium-level or high-level educated people [27]. To control for differences in the willingness for participation, a weighting factor provided by the Robert Koch Institute was used to approach the adult residential population structure in Germany.

We have shown above that our results agree with those of other countries. However, since there are considerable differences in health care systems and educational systems between other countries and Germany, international research and prevention programs can only be transferred to a limited extent. It is absolutely necessary to have national valid data in order to be able to establish precise public health interventions. One out of every two low-level educated adults aged 40–49 years in Germany is multimorbid hence the presence of multiple chronic conditions in one individual is very common. This is of high relevance, as for example, clinical recommendations still focus on single chronic diseases rather than dealing with multiple chronic conditions. Existing approaches in health care systems need to be complemented by enclosing information on risk factors and consequences of multimorbidity. Our findings with prevalence rates stratified by age and education represent contributing factors that should be considered within the development of prevention measures as well as programs for early detection of diseases in the public health sector in Germany.

The present study has analysed the association of multimorbidity, age and educational level but has not examined the relation between cause and effect. It may be possible that consequences of multimorbidity restrain the ability of young people to achieve a higher educational level. On the other hand, both, low educational qualification and being multimorbid, may be associated with poor lifestyle habits (e.g. smoking, alcohol, lack of exercise or excess weight). Multimorbidity is also associated with a higher mortality rate although it remains unclear to which extend the cumulative effects of coexisting diseases are responsible for an early death rather than functional disorder and mental disability related to the most severe disease. Hence, multimorbidity is a complex combination of effects and still not fully understood. Further research on multimorbidity is needed, in particular with regard to risk factors that seem to be associated with the early development of multiple chronic conditions in low level educated adults in Germany.

## Conclusions

Multimorbidity and its consequences are still a key challenge in public health systems. Our findings suggest that both, age and education are important aspects that have to be considered in the development of new prevention measures on multimorbidity. Existing single-disease approaches are increasingly inappropriate and new approaches covering the complex interactions of multiple chronic conditions are inevitable. Public health campaigns as well as programs for early detection of coexisting diseases in Germany especially have to focus on people ≤65 years with low educational qualification.

## Additional file

Additional file 1: Methodological details. The additional file provides methodological procedures in more detail. (PDF 260 kb)

## Abbreviations

ADM: Arbeitskreis Deutscher Markt- und Sozialforschungsinstitute e. V
BMI: Body mass index
CATI: Computer assisted telephone interviews
CI: Confidence interval
GEDA: German Health Update
ISCED: International Standard Classification of Education
OR: Odds ratio
PUF: Public Use File
WHO: World Health Organization
