# Supplementary material for: Prevalence of multimorbidity in Germany: impact of age and educational level in a cross-sectional study on 19,294 adults
Source: BMC Public Health. 2017 Oct 18;17:826. doi: 10.1186/s12889-017-4833-3 (PMC5648462; doi:10.1186/s12889-017-4833-3)
Supplement: Additional file 1: — Methodological details. The additional file provides methodological procedures in more detail. (PDF 260 kb) [file 12889_2017_4833_MOESM1_ESM.pdf]

**Supplemental material**

The sampling procedure [1, 2]

A two-stage sampling procedure was performed. For the selection at household level, the ADM-Sampling-System [3] based on the Gabler-Häder method [4] was used. The system covers all possible phone numbers in Germany, whether in use or not, and forms the target population. The total number of phone numbers was then randomly chosen provided that the total number equals eleven times the target number (the multiplication factor was estimated from previous surveys).

Random sampling at the individual level was performed using the Kish selection grid method [5]. Based on a private household, this method randomly selects an adult aged 18+ years out of all adults aged 18+ years in this private household.

Person flow [1, 2]

The target population comprised 364.206 telephone numbers. Of those, 19.294 respondents completed the interview while 61.362 individuals refused or broke off, 1.546 could not be contacted and 3.292 were excluded due to other reasons. In addition, 270.279 phone numbers were not eligible and for the remaining 8.433, eligibility was unknown (unknown if housing unit or unknown other).

The weighting procedure [1, 2]

The weighting factor was already provided by the federal public health institution Robert-Koch-Institute to adjust for the distribution of the sample by age, sex, level of education and residential region to match the German population.

According to the sampling design, the weighting factor combined the probability of selection (based on the household size and the number of different phone numbers in a household) and a factor related to region. Specifically, the weighting factor was calculated by

$$w_{k,i} = \frac{1}{pHH_{k,i} * t_{k,i}} * \frac{N_i}{\sum_{k=1}^{n_i} \left( \frac{1}{pHH_{k,i} * t_{k,i}} \right)}$$

with $pHH_{k,i}$: probability of selection of a respondent in household *k* in region *i*

$t_{k,i}$: number of phone numbers per household *k* in region *i*

$N_i$: population size in region *i*

$n_i$: number of interviews in region *i*

By post-stratification according to age, sex and education, the weighting factor was iteratively adjusted to match the distribution of different standard populations in Germany (data from the 2011 Census of the Federal Statistical Office and Microcensus 2011).

References


1. Robert-Koch-Institut. Beiträg zur Gesundheitsberichterstattung des Bundes: Daten und Fakten: Ergebnisse der Studie "Gesundheit in Deutschland aktuell 2012". 2014.
2. Lange C, Jentsch F, Allen J, Hoebel J, Kratz AL, Lippe E von der, et al. Data Resource Profile: German Health Update (GEDA)--the health interview survey for adults in Germany. Int J Epidemiol. 2015;44:442–50. doi:10.1093/ije/dyv067.
3. ADM Arbeitskreis Deutscher Markt- und Sozialforschungsinstitute e.V. The ADM-Sampling-System for Telephone Surveys. 2013.
4. Häder S, Gabler S. Ein neues Stichprobendesign für telefonische Umfragen in Deutschland. In: Gabler S, editor. Telefonstichproben in Deutschland. Opladen u.a.: Westdt. Verl.; 1998. p. 69–88. doi:10.1007/978-3-322-83334-1_6.
5. Kish L. A Procedure for Objective Respondent Selection within the Household. Journal of the American Statistical Association. 1949;44:380–7. doi:10.1080/01621459.1949.10483314.
